# Communicating Uncertainty in Written Consumer Health Information to the Public: Parallel-Group, Web-Based Randomized Controlled Trial

**DOI:** 10.2196/15899

**Published:** 2020-08-10

**Authors:** Roland B Büchter, Cornelia Betsch, Martina Ehrlich, Dennis Fechtelpeter, Ulrich Grouven, Sabine Keller, Regina Meuer, Constanze Rossmann, Andreas Waltering

**Affiliations:** 1 Institute for Quality and Efficiency in Health Care (IQWiG) Cologne Germany; 2 Media and Communication Science University of Erfurt Erfurt Germany

**Keywords:** uncertainty, consumer health information, decision making

## Abstract

**Background:**

Uncertainty is integral to evidence-informed decision making and is of particular importance for preference-sensitive decisions. Communicating uncertainty to patients and the public has long been identified as a goal in the informed and shared decision-making movement. Despite this, there is little quantitative research on how uncertainty in health information is perceived by readers.

**Objective:**

The aim of this study was to examine the impact of different uncertainty descriptions regarding the evidence for a treatment effect in a written research summary for the public.

**Methods:**

We developed 8 versions of a research summary on a fictitious drug for tinnitus with varying degrees (Q1), sources (Q2), and magnitudes of uncertainty (Q3). We recruited 2099 members of the German public from a web-based research panel. Of these, 1727 fulfilled the inclusion criteria and were randomly presented with one of these research summaries. Randomization was conducted by using a centralized computer with a random number generator. Web-based recruitment and data collection were fully automated. Participants were not aware of the purpose of the study and alternative presentations. We measured the following outcomes: perception of the treatment effectiveness (primary), certainty in the judgement of treatment effectiveness, perception of the body of evidence, text quality, and intended decision. The outcomes were self-assessed.

**Results:**

For the primary outcome, we did not find a global effect for Q1 and Q2 (*P*=.25 and *P*=.73), but we found a global effect for Q3 (*P*=.048). Pairwise comparisons showed a weaker perception of treatment effectiveness for the research summary with 3 sources of uncertainty compared to the version with 2 sources of uncertainty (*P*=.04). Specifically, the proportion of the participants in the group with 3 sources of uncertainty that perceived the drug as possibly beneficial was 9% lower than that of the participants in the group with 2 sources of uncertainty (92/195, 47.2% vs 111/197, 56.3%, respectively). The proportion of the participants in the group with 3 sources of uncertainty that considered the drug to be of unclear benefit was 8% higher than that of the participants in the group with 2 sources of uncertainty (72/195, 36.9% vs 57/197, 28.9%, respectively). However, there was no significant difference compared to the version with 1 source of uncertainty (*P*=.31). We did not find any meaningful differences between the research summaries for the secondary outcomes.

**Conclusions:**

Communicating even a large magnitude of uncertainty for a treatment effect had little impact on the perceived effectiveness. Efforts to improve public understanding of research are needed to improve the understanding of evidence-based health information.

**Trial Registration:**

German Clinical Trials Register DRKS00015911, https://www.drks.de/drks_web/navigate.do?navigationId=trial.HTML&TRIAL_ID=DRKS00015911

**International Registered Report Identifier (IRRID):**

RR2-10.2196/13425

## Introduction

Uncertainty pervades health care and is integral to evidence-informed decision making. Helping patients and consumers to understand and deal with uncertainty has been identified as one of the goals of the shared decision making and informed choices movement [1]. Understanding uncertainty is of particular importance for preference-sensitive decisions, that is, when there is a close trade-off between benefits and harms and patient values and preferences are highly variable. However, there are many difficulties in communicating uncertainty. Information providers often have to decide which of the many sources of uncertainty are the most relevant to patients. Examples include risk of bias, statistical uncertainty (imprecision), and lack of generalizability (indirectness). Selection is required to prevent information overload, which can prompt people to base their decisions on heuristics rather than on evidence [2]. Furthermore, communication of uncertainty may also have detrimental effects, for example, by hampering understanding or decreasing the credibility of the information provider [3,4].

Quantitative research on how to communicate uncertainty regarding the benefits and harms of treatments to patients and the public is limited. A systematic review by the Agency for Healthcare Research and Quality identified 8 controlled studies with 9 comparisons, including 6 randomized trials [5]. Four of these studies examined quantification of statistical uncertainty, 4 studied different ways of communicating net benefit, and 1 addressed uncertainty arising from the use of a surrogate outcome. These studies were very heterogeneous in terms of the context (cancer screening, treatment decision making, etc), interventions (written information, drug fact boxes, multifaceted interventions etc), and outcomes (risk perception, decision making, etc).

From our experience in providing evidence-based health information to the German public, enabling informed decisions often requires providing information on other types of uncertainty. For example, a patient with subacromial pain syndrome may wonder whether surgery using subacromial decompression may help with his/her condition. Two randomized, sham-controlled studies have shown no benefit of this treatment. However, few people in these trials had a hooked type III acromion. Thus, there is uncertainty whether this subgroup of patients may benefit from such a treatment. Whether such information is presented or not may impact readers in different ways and affect their choices. We are not aware of any studies investigating whether the perception of treatment effectiveness depends on the degree, type, and magnitude of uncertainty presented in written health information.

In order to test the main way in which we communicate uncertainty, we conducted a study addressing the following 3 questions.

Q1. Degree of uncertainty: Do members of the public perceive treatment effects differently depending on the certainty of the treatment effect (higher versus lower), which are expressed by different wordings (“studies show” or “studies indicate”)?

Q2. Type of uncertainty: Do members of the public interpret uncertain treatment effects differently depending on the type of uncertainty (publication bias, indirectness, imprecision)?

Q3. Number of sources of uncertainty: Is there an additive effect when multiple sources of uncertainty are presented (1-3 sources of uncertainty)?

We hypothesized that higher degrees of uncertainty expressed through different wordings or a larger magnitude of uncertainty reduce the perceived effectiveness of a treatment, the certainty in this judgement, and the intention to use the treatment. We further hypothesized that uncertainties due to vested interests/publication bias reduce perceived effectiveness to a larger extent than other sources of uncertainty. Lastly, we hypothesized that the ratings of text quality decrease with an increasing magnitude of uncertainty.

We investigated the 3 research questions by using 8 variations of a research summary for a fictitious drug for tinnitus that was set in a hypothetical treatment decision scenario. We designed the experiment as a randomized superiority study with 8 parallel groups allocated in an equal ratio (between-group design). This trial has been registered in the German Clinical Trials Register (DRKS00015911). The study protocol has been published elsewhere and includes the technical details of the study conduct [6].

## Methods

### Recruitment

We recruited members of the German public from a web-based research panel. Panel members were eligible if they were at least 18 years of age and able to read and write German. There were no other restrictions. We used a quota to ensure equal representation of different age groups (below and above 45 years) and sex. Once a quota cell was full, enrolment for this quota was closed.

### Study Procedure

After enrolment, the participants were provided with a short introduction to the study and an informed consent sheet. Panel members who agreed to participate were asked for information on age, sex, and educational degree based on the German school system (none/basic secondary/higher secondary/general entry qualification for university/university degree). We then asked them to imagine having tinnitus and having unsuccessfully tried several treatments. Participants were then randomly presented with one of the 8 versions of the research summary. These contained brief information on the medical condition and a short summary of the evidence for a fictitious new tinnitus medication (“Oroxil”). After the presentation of the research summaries, we collected data on the different outcomes by using a questionnaire developed for the purpose of this study. We allowed participants to refer to the research summary as needed while answering the questions. Before the conclusion of the experiment, participants were asked about their profession and history of tinnitus.

### Interventions

We developed 8 variations of the research summary based on standards and use of language in providing evidence-based health information to consumers through Germany’s statutory health website [7]. According to the objectives of the study, we systematically modified the research summary regarding the degree of uncertainty, the sources of uncertainty, and the magnitude of uncertainty. None of the other parts of the research summaries were altered between variations, including the numbers.

For Q1, we compared 3 alternative wordings for the expression of uncertainty. Version A describes a *certain* treatment effect and version B a *possible but not a certain* treatment effect (indication of effect). Version B1 was identical to B, but it included an additional statement on the *need for further research*. The wordings are based on the methods for the assessment of treatment benefits developed by the German Institute for Quality and Efficiency in Health Care (IQWiG) [8].

For Q2, we drew on the GRADE (Grading of Recommendations Assessment, Development, and Evaluation) framework to categorize different sources of uncertainty. According to GRADE, uncertainty can arise from risk of bias, (unexplained) inconsistency, indirectness (such as lack of generalizability or use of a surrogate outcome), imprecision, and other threats to validity such as publication bias or vested interests [9]. We therefore compared 3 additional research summaries describing publication bias/vested interests (B2), indirectness (B3), and imprecision (B4). We operationalized these sources of uncertainty by using everyday language (Table 1). We also included the variation B1 into this comparison.

For Q3, we compared a combination of 1, 2, or 3 sources of uncertainty (B4 vs B42 containing B4 and B2 vs B432 containing B4, B3, and B2).

This resulted in 8 variations of the research summary, 2 of which were used in 2 statistically independent comparisons (Table 1). An exemplary version of the research summary is provided in the Multimedia Appendix 1; this appendix shows the original German version that was translated into English for this publication. Multimedia Appendix 2 shows the original German version of the research summaries.

**Table 1:** Variations of the research summaries used to examine the 3 overarching research questions (translated from German).

Questions, Group identifier		Variations examined	Variations in the text
**Q1: Degree of uncertainty**			
	A	Effect shown	Studies show that Oroxil can reduce tinnitus.
	B	Indication of effect	Studies indicate that Oroxil may reduce tinnitus.
	B1	Indication of effect with general explanation	Studies indicate that Oroxil may reduce tinnitus. (…) The pros and cons of Oroxil cannot be fully judged, however. This requires further research.
**Q2: Type of uncertainty**			
	B1	Indication of effect with general explanation	As above
	B2	Publication bias/vested interests	Studies indicate that Oroxil may reduce tinnitus. (…) The pros and cons of Oroxil cannot be fully judged, however. The reason for this is that the company that developed the drug has not published all of the studies on Oroxil.
	B3	Indirectness (population)	Studies indicate that Oroxil may reduce tinnitus. (…) The pros and cons of Oroxil cannot be fully judged, however. The reason for this is that people who took part in the study were exposed to loud noises at work. It is uncertain whether the results also apply to other people.
	B4	Imprecision (small sample size)	Studies indicate that Oroxil may reduce tinnitus. (…) The pros and cons of Oroxil cannot be fully judged, however. The reason for this is that only a small number of people took part in the studies.
**Q3:** **Number of sources of uncertainty**			
	B4	Imprecision (small sample size)	As above
	B42	Publication bias/vested interests and imprecision	Studies indicate that Oroxil may reduce tinnitus. (…) The pros and cons of Oroxil cannot be fully judged, however. The reason for this is that only a small number of people took part in the studies. Furthermore, the company that developed the drug has not published all of the studies on Oroxil.
	B432	Publication bias/vested interests and imprecision and indirectness	Studies indicate that Oroxil may ease tinnitus. (…) The pros and cons of Oroxil cannot be fully judged, however. The reason for this is that the studies were small. Furthermore, people who took part in the study were exposed to loud noises at work. It is uncertain whether the results also apply to other people. Lastly, the company that developed the drug has not published all studies on Oroxil.

### Measurements

Our primary outcome was the perception of treatment effectiveness. Secondary outcomes were subjective certainty in the judgement of treatment effectiveness, perception of the body of evidence, intended decision, and perception of text quality. The outcomes were self-assessed through a web-based questionnaire. The outcome measures were developed and pretested by the author team if not stated otherwise.

The perception of treatment effectiveness was measured with 1 item on an ordinal scale (*How do you judge the effectiveness of Oroxil?*) with 5 possible answers: (1) it is proven that Oroxil can help, (2) Oroxil may possibly help, (3) it is unclear whether Oroxil helps, (4) Oroxil may not help, and (5) Oroxil definitely does not help.

Subjective certainty in the judgement of treatment effectiveness (*How certain do you feel in making this judgement?*) was measured on a 5-point Likert scale ranging from *not certain at all* to *very certain*. As this relates to the first question on the perception of treatment effectiveness, data on this item were gathered immediately after answering the first question.

The perception of the body of evidence was measured with a 6-item semantic differential scale with each item measured on a 5-point Likert scale. Participants were asked to rate the body of evidence as certain to uncertain, reliable to unreliable, valid to not valid, generalizable to not generalizable, excellent to poor, and trustworthy to untrustworthy.

The intended decision was measured using 1 item (*How would you decide?*) measured on a 5-point Likert scale with 2 poles: (1) definitely not take Oroxil and (2) definitely take Oroxil.

The perception of text quality was measured with a 9-item semantic differential scale based on previous literature [10,11]. The construct includes the following items measured on a 5-point Likert scale: interesting to uninteresting, balanced to 1-sided, comprehensible to incomprehensible, credible to incredible, clear to unclear, well done to not well done, professional to unprofessional, appealing to not appealing, and respectable to not respectable.

For the secondary outcomes *perception of the body of evidence* and *text quality,* we combined the items of each of the scales into 1 index by averaging their values, where a higher value indicates better perception of the body of evidence or text quality, respectively. The internal consistency was good with a Cronbach alpha of .81 for both indices.

We piloted a paper-and-pencil-version of the questionnaire and 2 versions of the research summary in a convenience sample of 40 students to test and optimize the reliability of the constructs, comprehensibility of the instructions, the stimuli, and the questions and to gather data for the sample size calculation.

### Randomization and Data Collection

Participants were recruited through a national web-based access panel run by a professional survey firm that provides incentives to participants (Bilendi). The data collection was run by the Survey Centre Bonn (uzbonn–Gesellschaft für empirische Sozialforschung und Evaluation), a spinout company of the Center for Evaluation and Methods at the University of Bonn. Unicom Intelligence survey software (formerly IBM SPSS Data Collection) was used for randomization and data collection [6]. Panel members were allocated after they had answered the demographic questions and were computer-checked for eligibility. As the experiment was web-based by using an automated and centralized computer system, the allocation sequence was concealed from the investigators and the data collectors. Participants were not aware of the purpose of the study and were not told about alternative presentations.

### Sample Size Calculation and Statistical Analysis

We calculated the sample size for the present study based on an effect size of Cohen *f*=0.15 derived from the pretest, a significance level of 5%, a power of 90%, and adjusted for use of a nonparametric test. This resulted in 159 participants per group. To allow some dropouts, we decided to randomize 1500 participants, equaling an average of 187.5 participants per group. The details of the sample size calculations are presented in the study protocol [6].

For the statistical analysis of the primary and secondary outcomes, we conducted Kruskal-Wallis tests to test for overall differences between the groups within each of our 3 primary study questions. We chose to use a nonparametric test to account for the types of scales used (ordinal scaling or unequal distances between items). In case of a significant overall difference, we conducted a multiple testing procedure to perform pairwise comparisons between the groups by means of the Dwass-Steel-Critchlow-Fligner multiple comparison analysis, which is based on pairwise 2-sample Wilcoxon comparisons. All statistical tests were 2-sided and performed using a 5% significance level. While we used nonparametric tests, data for the primary outcomes are presented as the proportion of responders for each possible answer and as means and standard deviations for the secondary outcomes in order to be more informative. To test the robustness of the findings, we repeated all the analyses by using analysis of variance (ANOVA). For this, we scored the primary outcome as a 5-point Likert scale in line with the other outcomes.

All participants were analyzed in the originally assigned group. Because we collected outcome data immediately after the presentation of the research summaries and because panel members were required to finish the questionnaire to receive their incentive, we had no major concerns regarding missing data. Therefore, we did not plan to employ any imputation methods and conducted all analyses on the data available. The statistical analysis was conducted by a statistician from the Medical Biometry Department at IQWiG with SAS version 9.4 (SAS Institute Inc).

As the experiment was web-based and participants came from a panel that provides incentives for participation, there was a risk that some participants would only participate to collect their incentives and not provide proper answers. As a measure of quality assurance, we excluded data from participants who answered all the questions in less than 2 minutes, spent less than 20 seconds on the page displaying the research summary, and spent less than 1.5 minutes between reading the research summary and completing the questionnaire (so-called speeders). These time limits were determined by test readings. We also excluded participants who provided the answers in the same row for the matrix questions, that is, when more than 1 item was displayed on the screen (so-called straightliners). This can be considered a conservative approach to exclude data by using a high threshold for implausibility. The exclusion of data from speeders and straightliners was planned a priori. We conducted sensitivity analyses by including these data to test the robustness of the main results.

### Ethical Review

A formal ethical approval for this study was waived by the institutional review board of the University of Erfurt owing to the negligible risk to the participants and because the study did not involve collection of identifiable data (EV-20180921). All participants gave their written informed consent to use and share their data for scientific purposes. Only anonymized nonidentifiable data that do not enable identification of the individual participants were collected and analyzed (Multimedia Appendix 3 shows the CONSORT-EHEALTH checklist).

## Results

### Sociodemographic Data

**Figure 1:**
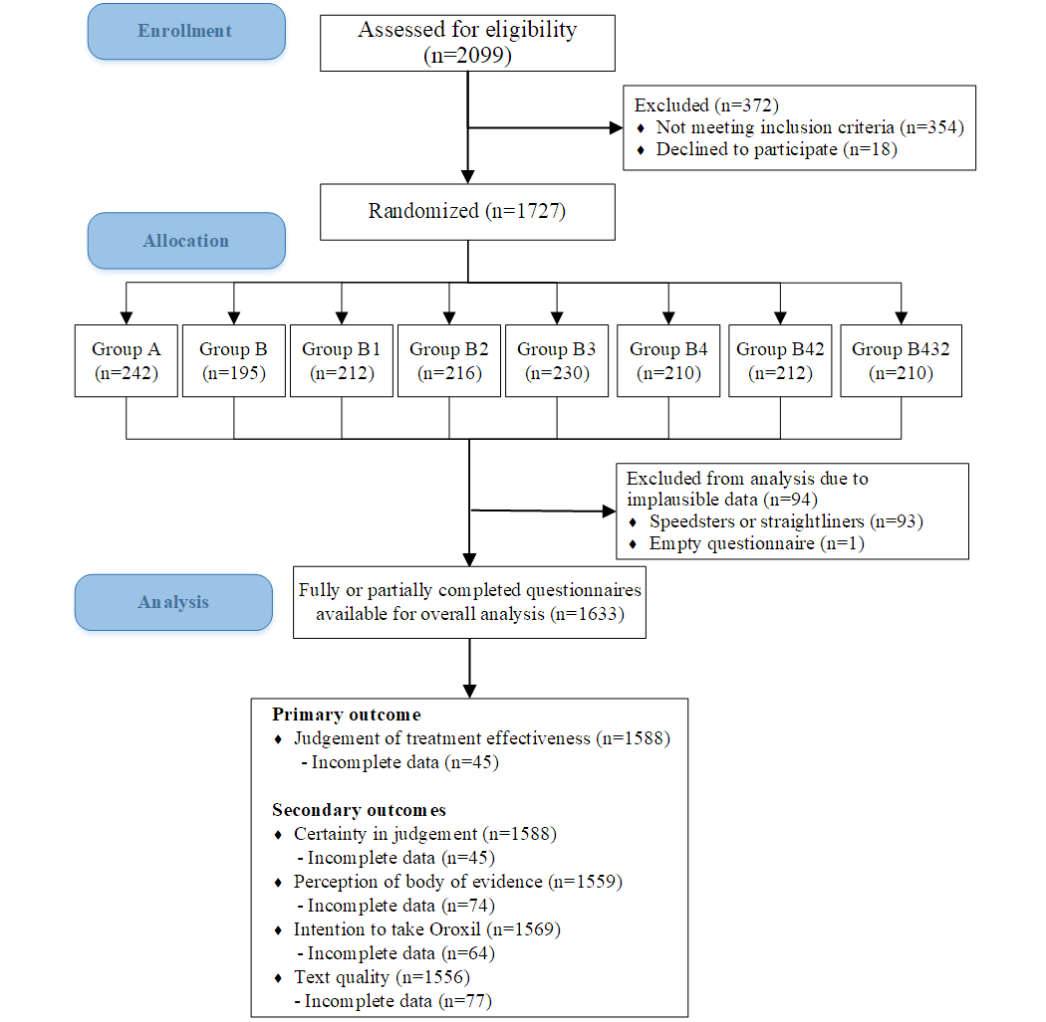
Study flow diagram.

A total of 2099 invited panel members were assessed for eligibility: 354 participants did not qualify owing to full quotas, 18 declined to participate, and 94 were excluded after the randomization because the data were deemed invalid (93 straightliners or speeders and 1 empty questionnaire). The sociodemographic characteristics of these participants excluded from the analysis owing to implausible data are presented in Multimedia Appendix 4. There is no visible pattern of postrandomization exclusions across groups (range 3%-7%). The final sample comprised 1633 participants. Data on the primary and secondary outcomes were available for 95%-97% of these participants, depending on the outcome (Figure 1). There were no notable differences between the number of participants with incomplete data among the groups (range 3%-8%). The number of participants randomized to each group and their sociodemographic characteristics are presented in Table 2.

**Table 2:** Sociodemographic characteristics of the participants.

Data		Group A^a^, n=242	Group B^b^, n=195	Group B1^c^, n=212	Group B2^d^, n=216	Group B3^e^, n=230	Group B4^f^, n=210	Group B42^g^, n=212	Group B432^h^, n=210
**Exclusions and missing data for the primary outcome, n (%)**									
	Excluded due to invalid data^i^	17 (7.0)	8 (4.1)	12 (5.7)	14 (6.5)	8 (3.5)	14 (6.7)	9 (4.2)	12 (5.7)
	Missing data^j^	3 (1.2)	6 (3.1)	7 (3.3)	12 (5.6)	3 (1.3)	5 (2.4)	3 (1.4)	6 (2.9)
**Analyzed, n (%)^j^**									
		222 (91.7)	181 (92.8)	193 (91.0)	190 (88.0)	219 (95.2)	191 (91.0)	197 (92.9)	195 (92.9)
**Demographic characteristics**									
	Mean age (years), (SD)	46 (13.7)	45 (13.5)	46 (13.3)	47 (13.5)	46 (13.7)	46 (13.9)	45 (13.9)	47 (14.2)
	Men, n (%)	105 (46.7)	91 (48.7)	90 (45.0)	103 (51.0)	106 (47.7)	101 (51.5)	96 (48.0)	117 (58.2)
	Women, n (%)	120 (53.3)	96 (51.3)	110 (55.0)	99 (49.0)	116 (52.3)	95 (48.5)	104 (52.0)	84 (41.8)
**Educational degree, n (%)**									
	None	0 (0)	1 (0.5)	1 (0.5)	2 (1.0)	0 (0)	0 (0)	0 (0)	0 (0)
	Basic secondary	30 (13.3)	23 (12.3)	30 (15.0)	23 (11.4)	27 (12.2)	23 (11.7)	23 (11.5)	29 (13.7)
	Higher secondary	74 (32.9)	73 (39.0)	65 (32.5)	82 (40.6)	67 (30.2)	63 (32.1)	80 (40.0)	68 (32.2)
	General entry qualification for university	54 (24.0)	44 (23.5)	48 (24.0)	44 (21.8)	68 (30.6)	48 (24.5)	46 (23.0)	68 (32.2)
	University degree	67 (29.8)	46 (24.6)	56 (28.0)	51 (25.2)	60 (27.0)	62 (31.6)	51 (25.5)	46 (21.8)
**History of tinnitus, n (%)^k^**									
	Currently symptomatic	31 (14.3)	30 (16.9)	25 (13.4)	22 (11.8)	22 (10.2)	23 (12.2)	27 (14.1)	30 (15.7)
	Previously symptomatic	37 (17.1)	21 (11.9)	27 (14.5)	37 (19.9)	28 (13.0)	33 (17.5)	26 (13.5)	24 (12.6)
	No history of tinnitus	149 (68.7)	126 (71.2)	134 (72.0)	127 (68.3)	166 (76.9)	133 (70.4)	139 (72.4)	137 (71.7)
**Profession, n (%)^k^**									
	Medical	17 (7.8)	13 (7.3)	20 (10.8)	19 (10.2)	18 (8.3)	18 (9.5)	26 (13.5)	18 (9.4)
	Nonmedical	200 (92.2)	164 (92.7)	166 (89.2)	167 (89.8)	198 (91.7)	171 (90.5)	166 (86.5)	173 (90.6)

^a^Effect shown.

^b^Indication of effect.

^c^Indication of effect with general explanation.

^d^Publication bias/vested interests.

^e^Indirectness (population).

^f^Imprecision (small sample size).

^g^Publication bias/vested interests and imprecision.

^h^Publication bias/vested interests and imprecision and indirectness.

^i^Speeders, straightliners, and empty questionnaires.

^j^Regarding primary outcome.

^k^Information not provided by 79 participants.

### Perception of the Treatment Effectiveness (Primary Outcome)

There were no overall statistical differences between the groups for Q1 (*degree of uncertainty*, *P*=.25). Numerically, the data show a slightly larger proportion of participants considering the drug to be of proven benefit in group A (*effect shown*) compared to that in group B (*indication of effect*) and B1 (*indication of effect and need for further research*), while a slightly smaller proportion of respondents considered it to be of unlikely benefit in group A (Table 3).

There were also no overall statistical differences between the groups for Q2 (*type of uncertainty*, *P*=.73). Numerically, condition B3 (*indirectness*) shifted some participants from considering the treatment to be clearly beneficial to considering it to be possibly beneficial (Table 3).

For Q3 (*magnitude of uncertainty*), there was a significant global effect on the perception of treatment effectiveness (*P*=.048). Pairwise comparisons showed a weaker perception of the effectiveness for the research summary with 3 sources of uncertainty compared to the version with 2 sources of uncertainty (*P*=.04). This is reflected in the answers: the proportion of the participants in the group with 3 sources of uncertainty that perceived the drug as possibly beneficial was 9% lower than that of the participants in the group with 2 sources of uncertainty (92/195, 47.2% vs 111/197, 56.3%, respectively), and the proportion of the participants in the group with 3 sources of uncertainty that considered the drug to be of unclear benefit was 8% higher than that of the participants in the group with 2 sources of uncertainty (72/195, 36.9% vs 57/197, 28.9%, respectively). Furthermore, with 3.6% (7/195) of the responses, the group with 3 sources of uncertainty was the only group with a considerable number of participants perceiving the drug as being unbeneficial (Table 3). However, there was no difference between the version with 1 and the version with 3 sources of uncertainty on pairwise comparison (*P*=.31). The results from the sensitivity analysis using ANOVA were consistent with those of the Kruskal-Wallis tests for all global comparisons. The results from the sensitivity analyses, including straightliners and speeders, were consistent with the main findings.

**Table 3:** Results of the primary outcome (perception of treatment effectiveness).

Questions (Q), Group identifier		Proportion of respondents’ answers, n (%)								*P* value^a^
		Benefit proven		Possible benefit	Benefit unclear		Benefit unlikely	No Benefit		
**Q1: Degree of uncertainty **										.25
	A^b^, n=222	33 (14.9)	115 (51.8)		63 (28.4)	10 (4.5)			1 (0.5)	
	B^c^, n=181	20 (11.1)	92 (50.8)		53 (29.3)	14 (7.7)			2 (1.1)	
	B1^d^, n=193	19 (9.8)	103 (53.4)		55 (28.5)	14 (7.3)			2 (1.0)	
**Q2: Type of uncertainty**										.73
	B1, n=193	19 (9.8)	103 (53.4)		55 (28.5)	14 (7.3)			2 (1.0)	
	B2^e^, n=190	23 (12.1)	96 (50.5)		59 (31.1)	12 (6.3)			0 (0.0)	
	B3^f^, n=219	13 (5.9)	124 (56.6)		64 (29.2)	16 (7.3)			2 (0.9)	
	B4^g^, n=191	16 (8.4)	99 (51.8)		60 (31.4)	15 (7.9)			1 (0.5)	
**Q3: Number of sources of uncertainty**										.048
	B4, n=191	16 (8.4)	99 (51.8)		60 (31.4)	15 (7.9)			1 (0.5)	
	B42^h^, n=197	17 (8.6)	111 (56.3)		57 (28.9)	12 (6.1)			0 (0.0)	
	B432^i^, n=195	12 (6.2)	92 (47.2)		72 (36.9)	12 (6.2)			7 (3.6)	

^a^Kruskal-Wallis test for global effect.

^b^Effect shown.

^c^Indication of effect.

^d^Indication of effect with general explanation.

^e^Publication bias/vested interests.

^f^Indirectness (population).

^g^Imprecision (small sample size).

^h^Publication bias/vested interests and imprecision.

^i^Publication bias/vested interests and imprecision and indirectness.

### Secondary Outcomes

The variations of the research summary had little impact on the secondary outcomes. There was a significant overall effect of the *type of uncertainty* on the perception of the body of evidence (*P*=.01). In pairwise comparison, the description for imprecision (B4) had a slightly larger effect on the *perceived limitations in the body of evidence* than the general statement that more research is needed (B1) (*P*=.01). Furthermore, there was a significant global effect on the *text quality* (*P*=.03). However, pairwise comparisons did not show statistical significance (*P*=.06 for the difference between B2 [*publication bias/vested interests*] and B4 [*imprecision*]). The results of the sensitivity analysis using ANOVA were consistent with those of the Kruskal-Wallis tests for the secondary outcomes. The results of the sensitivity analyses including straightliners and speeders were consistent with the main findings. The detailed results of the secondary outcomes are presented in Table 4.

**Table 4:** Results of the Kruskal-Wallis tests for global effects of the secondary outcomes.

Secondary outcome, mean (SD)	Q1: Degree of uncertainty				Q2: Type of uncertainty					Q3: Number of sources of uncertainty			
	A^a^	B^b^	B1^c^	*P* value	B1	B2^d^	B3^e^	B4^f^	*P* value	B4	B42^g^	B432^h^	*P* value
Certainty in judgement	3.60 (0.96)	3.49 (1.01)	3.49 (0.92)	.34	3.49 (0.92)	3.46 (0.92)	3.44 (0.93)	3.61 (0.98)	.22	3.61 (0.98)	3.47 (0.96)	3.49 (0.90)	.23
Perception of body of evidence	3.13 (0.76)	3.16 (0.81)	3.14 (0.77)	.80	3.14 (0.77)	3.00 (0.76)	3.00 (0.79)	2.89 (0.79)	.01	2.89 (0.79)	3.02 (0.75)	2.85 (0.68)	.09
Intention to take Oroxil	3.32 (1.18)	3.26 (1.18)	3.30 (1.23)	.91	3.30 (1.23)	3.26 (1.04)	3.26 (1.14)	3.24 (1.17)	.93	3.24 (1.17)	3.34 (1.05)	3.20 (1.10)	.51
Text quality	3.51 (0.75)	3.59 (0.80)	3.60 (0.80)	.33	3.60 (0.80)	3.44 (0.75)	3.58 (0.76)	3.60 (0.78)	.03	3.60 (0.78)	3.63 (0.70)	3.52 (0.68)	.23

^a^Effect shown.

^b^Indication of effect.

^c^Indication of effect with general explanation.

^d^Publication bias/vested interests.

^e^Indirectness (population).

^f^Imprecision (small sample size).

^g^Publication bias/vested interests and imprecision.

^h^Publication bias/vested interests and imprecision and indirectness.

## Discussion

### Summary of Results

For the primary outcome (*perception of effectiveness),* we did not find any statistically significant differences between alternative wordings for different *degrees of uncertainty* (Q1) and between different *sources of uncertainty* (Q2). However, there was a significant effect of the *number of sources of uncertainty* (Q3). As the differences between the groups in Q3 were small and the *P* value was just below the significance level, we believe that this result should still be interpreted cautiously. Furthermore, we did not see a dose-response effect of the number of sources of uncertainty on the perception of effectiveness. A possible explanation is that indirectness, which was added in the version of the research summary with 3 sources of uncertainty, is of particular importance to people. This seems plausible, as it is a tangible source of uncertainty, easy to understand, and relates to them personally. However, this type of uncertainty did not decrease the perception of treatment effectiveness in Q2 to a statistically significant degree.

The different presentations of uncertainty had no meaningful effect on the secondary outcomes, including the intention to take Oroxil. While the *body of evidence* was perceived as slightly more limited in the research summary describing imprecision, the difference between the groups was small and may be a result of the wordings used to measure the outcome (certain, reliable, etc).

### Possible Explanations

Psychological research suggests that discounting cues (eg, referring to a study with reduced credibility) prior to the provision of information can reduce the impact of that information [12]. Thus, our results may not apply to information that presents uncertainty earlier. If positioning had an effect, 1 possible solution in written health information would be to open the evidence section with a statement on the quality of the evidence and to make this as clear as possible, instead of presenting it at the end.

The results may also be explained by the neutral choice of language in the research summaries, thereby resulting in subtle differences between some of the variations. While stronger wordings may be needed to convey the uncertainties, we do not generally recommend the use of a more partial style of language as this would counteract the aims of providing balanced information and supporting informed decision making. This said, there are exceptions to this rule, for example, in case of highly implausible treatments such as homeopathy or new high-risk treatments lacking good evidence. On a more critical note, use of words may be unsuitable to convey the quality of evidence in general. While we identified little experimental research addressing similar questions, 1 study with physicians did not show an effect of different wordings such as “might” or “suggest” on conveying the strength of the recommendation within guidelines [13].

Lastly, it is possible that in a competitive choice situation, where there are 2 treatments, uncertainty information may matter more (ceteris paribus) because it helps to discriminate drugs from each other. This may be particularly true for the outcome intended treatment decision, where, in our study, participants in all groups tended to opt for taking the drug. A possible explanation for the lack of differences between the groups is that the scenario suggested that there are no other proven treatments for tinnitus.

On an average, 52.4% (832/1588) of all the participants considered the treatment to be possibly helpful, and about 30.4% (483/1588) considered the benefit of the drug to be unclear, including the group without any expressed uncertainty (A). A possible explanation for this result is that the use of numbers to communicate the response rate for the treatment may have diluted the effects of the uncertainty descriptions. This raises the question of whether communicating certainty in the quality of evidence in parallel with probability information is the actual challenge for communicators rather than communicating uncertainty. The explanation seems likely since the public is still not used to such presentations and probably has difficulties in distinguishing between uncertainty from limitations in the quality of evidence (the certainty in an effect estimate) and uncertainty in terms of dealing with probabilistic outcomes (the magnitude of an effect). This has been shown in a qualitative study conducted to improve Cochrane plain language summaries [14]. Users ignored or reduced the 4 grading levels (very low to high) that were used in the plain language summaries, irrespective of the choice of words used to delineate uncertainty. An important difference between that study and ours is that we looked at uncertainties from specific sources, whereas Glenton and colleagues evaluated modified GRADE summary of findings tables without making the sources of uncertainty explicit to the users. In a follow-up randomized trial, one of the enhanced plain-language summaries appeared to improve understanding of the quality of evidence [15]. However, the format is limited to reviews using the GRADE system or requires health information developers to apply the GRADE framework. Furthermore, the way understanding was measured in this study was problematic. Specifically, the use of catchphrases in the enhanced plain language summary may have led more participants to answer correctly to the questions rather than improved understanding over the conventional format. A focus group study of a decision aid of management options for women with breast cancer and *BRCA*-positive gene mutations looked at ribbons to illustrate levels of evidence (4 grades from bronze to platinum). In this study, the users assumed that if data are presented using numbers and graphs, people will automatically consider them to be of sufficient quality [16]. These explanations aside, people may simply tend to focus on effectiveness when reading health information and often overestimate the benefits of treatments [17].

### Possible Solutions

A possible but, in many cases, an inappropriate solution would be to avoid the use of numbers. Presenting quantitative information on treatment effects has become a standard requirement for evidence-based health information and decision aids because probabilities on the benefits and harms support realistic expectations and allow patients to weigh the pros and cons [18]. Furthermore, words have been shown to be unsuitable to express probabilities in several studies [19,20]. Instead, it might be necessary to provide readers with a clear statement on what a specific type of uncertainty means in order to aid interpretation. In a randomized trial on drug fact boxes that studied uncertainty due to the use of surrogate outcomes and unknown safety profiles for newly approved drugs, both directive and nondirective explanations on what these uncertainties mean changed the intention to take the drug [21]. However, the effects were relatively small, with an absolute change of 12% and 19%. While this appears to be a promising approach, the implications of the uncertainties arising from different populations, publication bias, and small sample sizes as tested in our study can be ambiguous and require a high level of judgement. Thus, providing such explanations may be difficult in written health information aimed at a broad range of readers in some cases.

Lastly, providers of evidence-based health information should carefully consider whether the quality of the evidence is sufficient for an effect to be presented at all. While fine-graded levels of evidence such as in the GRADE system are useful to health care professionals, they may be too nuanced for patients and the public. Instead, it may be more sensible to use a higher cut-off for presenting treatment effects (eg, at least moderate quality of evidence within GRADE) while labelling low or very low quality of evidence to be unclear. This seems reasonable as the true effect may substantially differ from the study estimate for evidence of low or very low quality according to the current definition within the GRADE system [22].

While there are many possible solutions to dealing with uncertainty, it is unlikely that one will fit all scenarios in practice. How uncertainty is presented may depend on many factors such as the format and aims of the health information, the target group, the severity of the condition, the risks of the intervention, and the availability and number of treatment options. Thus, and maybe most importantly, the judgements made in this process should be carefully reflected.

### Strengths and Limitations

The main strengths of our study are the large sample population based on a sample size calculation for a conservative effect, few missing data, and a robust protocol-driven methodology. Furthermore, we used a research summary that is concordant with the current standards of evidence-based health information, including the use of probabilities to communicate benefits and harms in order to allow weighing the pros and cons. The weaknesses include the use of a hypothetical scenario and restriction to 1 (relatively benign) condition and treatment. Furthermore, the choice of the size of the presented treatment effects is debatable. We decided to use a small absolute effect since large treatment effects are rare in practice [23]. In our experience, they often range from around 0.1% to 1% risk difference for primary preventive or screening interventions (small) to about 5% (medium) to 20% (large) for symptom improvement. Thus, we believe that our information provides a good representation of what developers of evidence-based health information are confronted with in practice. A possible limitation to using simple frequencies is that readers may mistake them for sample size. We cannot exclude that this had an impact on their interpretation of the research summaries. Another limitation is that this study was conducted in Germany and these results may not be generalizable to other countries. Finally, the way the primary outcome was measured may not have been sensitive enough to capture differences and there is always a risk that participants tend to avoid more extreme answers. However, the lack of meaningful effects on secondary outcomes supports the notion that there are indeed few perceived differences between the research summaries. Thus, it seems unlikely that measuring perceived treatment effectiveness using a visual analog or Likert scale, for example, would have made a difference to the results. The downside of such measurements is that the relevance of the results is difficult to interpret. Owing to these limitations, we cannot completely rule out that participants are insensitive to uncertainty information.

### Recommendations for Future Research

In order for uncertainty descriptions to be useful in conveying limitations in the quality of evidence, the public has to be aware that not all research is of equal quality and that the size of an effect and the confidence in the effect are separate concepts. Therefore, it would be helpful to develop or enhance and implement interventions that explain basic research concepts and skills. A notable recent effort in this direction has been the Key Concepts for Informed Health Choices Project [24-26]. It may also be helpful to develop more radical ways of presenting uncertainty together with consumers. Future studies could also assess the effect of uncertainty information in situations wherein patients decide between several treatments. In terms of the methods, future studies could use factorial designs to study different combinations of uncertainty descriptions.

### Conclusion

In conclusion, providers of written evidence-based health information or decision aids should not assume that consumers and patients will understand the intentions and meaning of uncertainty descriptions without further explanation or support. The most likely explanation for this is that the public does not distinguish between the concepts of quality of evidence and magnitudes of effect. The challenge in communicating uncertainty should not be used as an argument to withhold uncertainty information from patients and the public. Future research should aim to understand how the public or patients process information regarding limitations of evidences in order to understand why the degree and types of uncertainty seem to have little impact on the perceptions of effectiveness or treatment intentions.

## Appendix

Multimedia Appendix 1 Exemplary research summary.

Multimedia Appendix 2 German versions of the research summaries.

Multimedia Appendix 3 CONSORT-EHEALTH checklist.

Multimedia Appendix 4 Sociodemographic characteristics of the excluded participants.

## Abbreviations

ANOVA: analysis of variance
GRADE: Grading of Recommendations Assessment, Development and Evaluation
IQWiG: German Institute for Quality and Efficiency in Health Care

## References

[1] Institute of Medicine (US) Committee on Quality of Health Care in America (2001). Crossing the Quality Chasm: A New Health System for the 21st Century.

[2] Gigerenzer G, Gaissmaier W (2011). Heuristic decision making. Annu Rev Psychol.

[3] Longman T, Turner RM, King M, McCaffery KJ (2012). The effects of communicating uncertainty in quantitative health risk estimates. Patient Educ Couns.

[4] Sladakovic J, Jansen J, Hersch J, Turner R, McCaffery K (2016). The differential effects of presenting uncertainty around benefits and harms on treatment decision making. Patient Educ Couns.

[5] McCormack L, Sheridan S, Lewis M, Boudewyns V, Melvin CL, Kistler C, Lux LJ, Cullen K, Lohr KN (2013). Communication and dissemination strategies to facilitate the use of health-related evidence. Evid Rep Technol Assess (Full Rep).

[6] Büchter Roland Brian, Betsch C, Ehrlich M, Fechtelpeter D, Grouven U, Keller S, Meuer R, Rossmann C, Waltering A (2019). Communicating Uncertainty From Limitations in Quality of Evidence to the Public in Written Health Information: Protocol for a Web-Based Randomized Controlled Trial. JMIR Res Protoc.

[7] What we do at InformedHealth.org. Informed Health Online.

[8] Institute for Quality and Efficiency in Health Care (IQWiG) (2017). General Methods: Version 6.0.

[9] Guyatt GH, Oxman AD, Vist GE, Kunz R, Falck-Ytter Y, Alonso-Coello P, Schünemann Holger J, GRADE Working Group (2008). GRADE: an emerging consensus on rating quality of evidence and strength of recommendations. BMJ.

[10] Brosius H, Birk M (1994). Text-Bild-Korrespondenz und Informationsvermittlung durch Fernsehnachrichten. Rundfunk und Fernsehen.

[11] Gehrau V (2019). Fernsehbewertung Und Fernsehhandlung.

[12] Kumkale GT, Albarracín Dolores (2004). The sleeper effect in persuasion: a meta-analytic review. Psychol Bull.

[13] Akl EA, Guyatt GH, Irani J, Feldstein D, Wasi P, Shaw E, Shaneyfelt T, Levine M, Schünemann Holger J (2012). "Might" or "suggest"? No wording approach was clearly superior in conveying the strength of recommendation. J Clin Epidemiol.

[14] Glenton C, Santesso N, Rosenbaum S, Nilsen ES, Rader T, Ciapponi A, Dilkes H (2010). Presenting the results of Cochrane Systematic Reviews to a consumer audience: a qualitative study. Med Decis Making.

[15] Santesso N, Rader T, Nilsen ES, Glenton C, Rosenbaum S, Ciapponi A, Moja L, Pardo JP, Zhou Q, Schünemann Holger J (2015). A summary to communicate evidence from systematic reviews to the public improved understanding and accessibility of information: a randomized controlled trial. J Clin Epidemiol.

[16] Culver JO, MacDonald DJ, Thornton AA, Sand SR, Grant M, Bowen DJ, Burke H, Garcia N, Metcalfe KA, Weitzel JN (2011). Development and evaluation of a decision aid for BRCA carriers with breast cancer. J Genet Couns.

[17] Hoffmann TC, Del Mar C (2015). Patients' expectations of the benefits and harms of treatments, screening, and tests: a systematic review. JAMA Intern Med.

[18] Trevena LJ, Zikmund-Fisher BJ, Edwards A, Gaissmaier W, Galesic M, Han PKJ, King J, Lawson ML, Linder SK, Lipkus I, Ozanne E, Peters E, Timmermans D, Woloshin S (2013). Presenting quantitative information about decision outcomes: a risk communication primer for patient decision aid developers. BMC Med Inform Decis Mak.

[19] Blalock SJ, Sage A, Bitonti M, Patel P, Dickinson R, Knapp P (2016). Communicating information concerning potential medication harms and benefits: What gist do numbers convey?. Patient Educ Couns.

[20] Büchter Roland Brian, Fechtelpeter D, Knelangen M, Ehrlich M, Waltering A (2014). Words or numbers? Communicating risk of adverse effects in written consumer health information: a systematic review and meta-analysis. BMC Med Inform Decis Mak.

[21] Schwartz LM, Woloshin S (2011). Communicating uncertainties about prescription drugs to the public: a national randomized trial. Arch Intern Med.

[22] Balshem H, Helfand M, Schünemann Holger J, Oxman AD, Kunz R, Brozek J, Vist GE, Falck-Ytter Y, Meerpohl J, Norris S, Guyatt GH (2011). GRADE guidelines: 3. Rating the quality of evidence. J Clin Epidemiol.

[23] Kent DM, Trikalinos TA (2009). Therapeutic innovations, diminishing returns, and control rate preservation. JAMA.

[24] Castle JC, Chalmers I, Atkinson P, Badenoch D, Oxman AD, Austvoll-Dahlgren A, Nordheim L, Krause LK, Schwartz LM, Woloshin S, Burls A, Mosconi P, Hoffmann T, Cusack L, Albarqouni L, Glasziou P (2017). Establishing a library of resources to help people understand key concepts in assessing treatment claims-The "Critical thinking and Appraisal Resource Library" (CARL). PLoS One.

[25] Chalmers I, Oxman AD, Austvoll-Dahlgren A, Ryan-Vig S, Pannell S, Sewankambo N, Semakula D, Nsangi A, Albarqouni L, Glasziou P, Mahtani K, Nunan D, Heneghan C, Badenoch D (2018). Key Concepts for Informed Health Choices: a framework for helping people learn how to assess treatment claims and make informed choices. BMJ Evid Based Med.

[26] Cusack L, Del Mar CB, Chalmers I, Gibson E, Hoffmann TC (2018). Educational interventions to improve people's understanding of key concepts in assessing the effects of health interventions: a systematic review. Syst Rev.

