# Supplementary material for: Communicating Uncertainty in Written Consumer Health Information to the Public: Parallel-Group, Web-Based Randomized Controlled Trial
Source: J Med Internet Res. 2020 Aug 10;22(8):e15899. doi: 10.2196/15899 (PMC7445603; doi:10.2196/15899)
Supplement: Multimedia Appendix 1 [file jmir_v22i8e15899_app1.docx]

**Multimedia Appendix 1**

**1) Introduction**

**Please imagine the following situation:**

You have had tinnitus for a while now. Tinnitus leads to bothersome noises in the ear such as ringing, hissing, buzzing or roaring. The symptoms can last for months or years. In many people, its causes remain unclear, which makes tinnitus difficult to treat.

You have tried a number of different treatments, none of which has helped. Recently, a new drug has appeared on the market. You decide to inform yourself about the benefits of this drug. We ask you to read the following information carefully and answer a few questions regarding the text.

**2) Exemplary research summary (Variation A):**

**Treatments for tinnitus**

Several treatments are offered and used for tinnitus. None of them is particularly effective, however. A new drug called Oroxil has recently become available.

Oroxil improves the blood flow to the inner ear. This is hoped to reduce tinnitus noises or get rid of them completely. Oroxil is taken as a tablet once a day.

**What are the pros and cons of Oroxil?**

Studies show that Oroxil can reduce tinnitus symptoms. In these studies, one half of the participants took the drug, the other half a placebo (fake drug). After six months, the groups were compared. The result:

- Without treatment, ear noises significantly improved in about 20 out of 100 people.
- With treatment, ear noises significantly improved in about 25 out of 100 people.

In other words, Oroxil improved tinnitus symptoms significantly in an extra 5 out of 100 people after 6 months.

The drug lead to minor side effects such as occasional dizziness or tiredness in 3 out of 100 people. No severe side effects occurred.
