# Supplementary material for: Communicating Uncertainty in Written Consumer Health Information to the Public: Parallel-Group, Web-Based Randomized Controlled Trial
Source: J Med Internet Res. 2020 Aug 10;22(8):e15899. doi: 10.2196/15899 (PMC7445603; doi:10.2196/15899)
Supplement: Multimedia Appendix 2 [file jmir_v22i8e15899_app2.docx]

**Multimedia Appendix 2**

**Original German Version of the research summary**

**1) Introduction**

Versetzen Sie sich bitte in folgende Situation:

Sie haben seit längerem einen Tinnitus. Ein Tinnitus führt zu belastenden Ohrgeräuschen wie Klingeln, Pfeifen, Summen oder Brummen. Die Beschwerden können über Monate oder Jahre anhalten. Bei vielen Menschen bleibt die Ursache für die Ohrgeräusche unbekannt. Dies erschwert eine gezielte Behandlung.

Sie haben schon verschiedene Behandlungen ausprobiert, von denen keine geholfen hat. Seit kurzem ist ein neues Medikament auf dem Markt. Sie informieren sich nun über den Nutzen dieses Medikaments. Wir bitten Sie, die folgende Information gründlich durchzulesen und anschließend einige Fragen zu dem Text zu beantworten.

**2) Exemplary research summary (Variation A)**

**Behandlung von Tinnitus**

Gegen chronischen Tinnitus werden zahlreiche Behandlungen angeboten und eingesetzt. Bislang gibt es kaum wirksame Therapien. Seit kurzem steht das Medikament Oroxil zur Verfügung.

Oroxil verbessert die Durchblutung im Innenohr. Dadurch soll es die Ohrgeräusche lindern oder ganz zum Verschwinden bringen. Oroxil wird einmal täglich als Tablette eingenommen.

**Welche Vor- und Nachteile hat Oroxil?**

Studien zeigen, dass Oroxil Tinnitus lindern kann. In den Studien nahm die Hälfte der Teilnehmenden das Medikament ein, die andere Hälfte ein Scheinmedikament (Placebo). Nach sechs Monaten wurden die beiden Gruppen miteinander verglichen. Das Ergebnis:

- Bei etwa 20 von 100 Menschen, die ein Placebo einnahmen, waren die Ohrgeräusche deutlich abgeklungen.
- Bei etwa 25 von 100 Menschen, die Oroxil einnahmen, waren die Ohrgeräusche deutlich abgeklungen.

In anderen Worten: Nach sechs Monaten hatte Oroxil die Tinnitus-Beschwerden bei zusätzlich 5 von 100 Menschen deutlich gelindert.

Oroxil führte bei etwa 3 von 100 Menschen zu Nebenwirkungen wie gelegentlichem Schwindel oder Müdigkeit. Schwere Nebenwirkungen traten nicht auf.

**Variations used for the original German versions of the research summaries**

| **Nr.** | **Version** | **Umsetzung im Text** |
| --- | --- | --- |
| **Varianten für Fragestellung 1** | | |
| A | Beleg | Studien zeigen, dass Oroxil Tinnitus lindern kann. |
| B | Hinweis | Studien deuten darauf hin, dass Oroxil Tinnitus lindern kann. |
| B1 | Hinweis mit allgemeiner Erklärung. | Studien deuten darauf hin, dass Oroxil Tinnitus lindern kann. […]  Die Vor- und Nachteile lassen sich aber noch nicht sicher beurteilen. Hierfür sind weitere Studien notwendig. |
| **Varianten für Fragestellung 2** | | |
| B1 | Hinweis mit allgemeiner Erklärung. | Siehe oben |
| B2 | Hinweis plus Publikations-Bias | Studien deuten darauf hin, dass Oroxil Tinnitus lindern kann. […]  Die Vor- und Nachteile lassen sich aber noch nicht sicher beurteilen. Der Grund: *Der Hersteller hat nicht alle dazu durchgeführten Studien veröffentlicht.* |
| B3 | Hinweis plus Übertragbarkeit | Studien deuten darauf hin, dass Oroxil Tinnitus lindern kann. […]  Die Vor- und Nachteile lassen sich aber noch nicht sicher beurteilen. Der Grund: *An den Studien nahmen nur Personen teil, die am Arbeitsplatz einer erhöhten Lärmbelästigung ausgesetzt waren. Es ist ungewiss, ob die Ergebnisse auch für andere Betroffene gelten.* |
| B4 | Hinweis plus statistische Unsicherheit (Fallzahlgröße) | Studien deuten darauf hin, dass Oroxil Tinnitus lindern kann. […]  Die Vor- und Nachteile lassen sich aber noch nicht sicher beurteilen. Der Grund: *An den Studien nahmen nur wenige Personen teil.* |
| **Varianten für Fragestellung 3** | | |
| B4 | Hinweis plus statistische Unsicherheit (Fallzahlgröße) | Siehe oben |
| B42 | Hinweis plus statistische Unsicherheit plus Publikations-Bias | Studien deuten darauf hin, dass Oroxil Tinnitus lindern kann. […]  Die Vor- und Nachteile lassen sich aber noch nicht sicher beurteilen. Der Grund: *An den Studien nahmen nur wenige Personen teil. Zudem hat der Hersteller des Medikaments nicht alle dazu durchgeführten Studien veröffentlicht.* |
| B432 | Hinweis plus statistische Unsicherheit plus Publikations-Bias plus Übertragbarkeit | Studien deuten darauf hin, dass Oroxil Tinnitus lindern kann. […]  Die Vor- und Nachteile lassen sich aber noch nicht sicher beurteilen. Der Grund: *An den Studien nahmen nur wenige Personen teil. Diese waren zudem am Arbeitsplatz einer erhöhten Lärmbelastung ausgesetzt Es ist deshalb ungewiss, ob die Ergebnisse so auch für andere Betroffene gelten. Außerdem hat der Hersteller des Medikaments nicht alle dazu durchgeführten Studien veröffentlicht.* |
