# Supplementary material for: Communicating Uncertainty in Written Consumer Health Information to the Public: Parallel-Group, Web-Based Randomized Controlled Trial
Source: J Med Internet Res. 2020 Aug 10;22(8):e15899. doi: 10.2196/15899 (PMC7445603; doi:10.2196/15899)
Supplement: Multimedia Appendix 4 [file jmir_v22i8e15899_app4.doc]

**Multimedia appendix 3**

**Supplementary table: sociodemographic characteristics of participants excluded due to implausible data**

|  |  | **Group A** | **Group B** | **Group B1** | **Group B2** | **Group B3** | **Group B4** | **Group B42** | **Group B432** |
| --- | --- | --- | --- | --- | --- | --- | --- | --- | --- |
| **N (%)** |  | 17 (7.0) | 8  (4.1) | 12 (5.7) | 14 (6.5) | 8  (3.5) | 14 (6.7) | 9  (4.2) | 12 (5.7) |
| **Demographic characteristics** |  |  |  |  |  |  |  |  |  |
|  | mean age (years), (SD) | 35 (9.8) | 44 (9.0) | 36 (8.9) | 36 (16.7) | 43 (9.5) | 42 (12.7) | 45 (10.3) | 37 (12.9) |
|  | Men, n | 7 | 6 | 8 | 6 | 4 | 7 | 7 | 7 |
|  | Women, n | 10 | 2 | 4 | 8 | 4 | 7 | 2 | 5 |
| **Educational degree, n** |  |  |  |  |  |  |  |  |  |
|  | None | 0 | 0 | 2 | 1 | 0 | 0 | 0 | 0 |
|  | basic secondary | 1 | 1 | 2 | 2 | 0 | 2 | 2 | 3 |
|  | higher secondary | 10 | 2 | 2 | 1 | 4 | 6 | 4 | 4 |
|  | general entry qualification for university | 1 | 4 | 4 | 4 | 2 | 5 | 2 | 3 |
|  | university degree | 5 | 1 | 2 | 6 | 2 | 1 | 1 | 2 |
| **History of tinnitus, n** |  |  |  |  |  |  |  |  |  |
|  | Currently symptomatic | 3 | 1 | 0 | 2 | 0 | 0 | 1 | 1 |
|  | Previously symptomatic | 2 | 2 | 6 | 2 | 1 | 3 | 3 | 0 |
|  | No history of tinnitus | 12 | 5 | 6 | 10 | 7 | 11 | 5 | 11 |
| **Profession, n** |  |  |  |  |  |  |  |  |  |
|  | Medical | 4 | 2 | 2 | 4 | 0 | 3 | 2 | 1 |
|  | Non-medical | 13 | 6 | 10 | 10 | 8 | 11 | 7 | 11 |

SD=standard deviation
